# Robotic quantification of upper extremity loss of independent joint control or flexion synergy in individuals with hemiparetic stroke: a review of paradigms addressing the effects of shoulder abduction loading

**DOI:** 10.1186/s12984-016-0203-0

**Published:** 2016-10-29

**Authors:** Michael D. Ellis, Yiyun Lan, Jun Yao, Julius P. A. Dewald

**Affiliations:** 1 Department of Physical Therapy and Human Movement Sciences, Feinberg School of Medicine, Northwestern University, 645 N. Michigan Ave. Suite 1100, Chicago, IL 60611 USA; 2 Department of Biomedical Engineering, McCormick School of Engineering, Northwestern University, Chicago, IL USA; 3 Department of Physical Medicine and Rehabilitation, Feinberg School of Medicine, Northwestern University, Chicago, IL USA

**Keywords:** Stroke, Rehabilitation, Robotics, Arm, Flexion synergy, Loss of independent joint control, Outcome, Reaching, Function

## Abstract

Unsupported or “against-gravity” reaching and hand opening movements are greatly impaired in individuals with hemiparetic stroke. The reduction in reaching excursion and hand opening is thought to be primarily limited by abnormal muscle co-activation of shoulder abductors with distal limb flexors, known as *flexion synergy*, that results in a loss of independent joint control or joint individuation. Our laboratory employs several methods for quantifying this movement impairment, however the most documented techniques are sophisticated and laboratory-based. Here a series of robotic methods that vary in complexity from comprehensive (laboratory-based) to focused (clinically relevant) are outlined in detail in order to facilitate translation and make recommendations for utilization across the translational spectrum as part of Journal of NeuroEngineering and Rehabilitation thematic series, “Technically-advanced assessments in sensory motor rehabilitation.” While these methods focus on our published work utilizing the device, ACT^3D^, these methods can be duplicated using any mechatronic device with the appropriate characteristics. The common thread and most important aspect of the methods described is addressing the deleterious effects of abduction loading. Distal upper extremity joint performance is directly and monotonically modulated by proximal (shoulder abduction) joint demands. The employment of robotic metrics is the best tool for selectively manipulating shoulder abduction task requirements spanning the individual’s full range of shoulder abduction strength. From the series of methods and the concluding recommendations, scientists and clinicians can determine the ideal robotic quantification method for the measurement of the impact of loss of independent joint control on reaching and hand function.

## Background

### Purpose

*This work was developed as part of the project “State of the Art Robot-Supported assessments (STARS)” in the frame of the COST Action TD1006 “European Network on Robotics for NeuroRehabilitation* [1]*.” The goal of STARS is to give neurorehabilitation clinical practitioners and scientists recommendations for the development, implementation, and administration of different indices of robotic assessments, grounded on scientific evidence.*

The formatting of this manuscript employs a standardized structure as part of the thematic series, “Technically-advanced assessments in Sensory Motor Rehabilitation.” The manuscript provides a detailed description of experimental paradigms in order to facilitate standardized replication and translation to clinical practice and research. Following the brief introduction, the operational definition is provided for *“loss of independent joint control,”* the contextual motor impairment of individuals with stroke discussed in the manuscript. Subsequent sections review robotic methods developed in our laboratory used to quantify the effect of loss of independent joint control on reaching and hand function. The methods discussed progress from well-documented laboratory-based paradigms to suggestions for expedited and clinically relevant methods. Finally, concluding remarks offer recommendations for choosing the appropriate metric based upon relevant constraints across the translational spectrum including the level of detail required, time constraints for measurement, and devices available to the scientist/clinician.

### Context

Residual motor system impairments limit the activities conducted in daily life and restrict participation in life roles in individuals with moderate to severe chronic stroke. Two primary motor system impairments characterizing classic hemiparesis are muscle weakness and abnormal stereotypical movements/synergies. Contemporary clinical tools attempt to evaluate these motor impairments however are limited by subjective/nominal scoring descriptors and/or observational methods and are confounded by the interdependency of these phenomena. For example, following stroke there is a relative weakness on the paretic side in that the production of joint torque at a single joint is less that on the non-paretic side. However, when the individual is required to first produce shoulder abduction torque, abnormal co-activation with elbow flexors occurs with greater abduction torque production [2], resulting in a task dependent weakness of elbow extension [3, 4]. This abnormal co-activation of shoulder abductors with distal limb flexors [2] was described observationally as stereotypical movements and labeled *flexion synergy* [5, 6] in the mid 20th century. Early quantitative dynamic movement studies of hemiparetic reaching demonstrated the same phenomena showing that outward reaching magnitude is dramatically reduced when required to support the arm against gravity as compared to when supported on a frictionless table [7] and is not predicted by muscle weakness [8]. These studies demonstrated that two fundamental motor system impairments could be independently and quantitatively evaluated but required more sophisticated equipment than available in clinical practice. The application of robotics, as presented in this manuscript, offers a means to account for the presence of weakness/paresis and systematically quantify the impact of flexion synergy on reaching function through kinetic/kinematic measurement.

## Definition of loss of independent joint control

Individuals with moderate to severe hemiparetic stroke exhibit compromised upper extremity function due in part to a loss of independent joint control. Neuromechanistically, this phenomena is thought to be due to an increased reliance on contralesional corticoreticulospinal motor pathways (see [9] for review). Early quantitative movement analysis studies described the phenomena as disturbed limb dynamics [10] and disrupted interjoint coordination [11] that was observed during reaching movements. Of specific relevance to the evaluation with rehabilitation robotics is that the abnormal co-activation of distal limb flexors with proximal shoulder abductors is task-dependent [3, 12, 13] and dynamic [7, 14, 15], meaning that the more one attempts to drive the limb, the greater the activation of the flexion pattern and the lessor the ability to move outside of this pattern such as during a reach against gravity. A device with the capability of progressively manipulating proximal joint requirements is needed to fully describe the effects of loss of independent joint control on reaching and hand function in hemiparesis [14].

- Therefore, the operational definition of *loss of independent joint control* is: *the dynamic and task-dependent reduction of joint individuation due to proximal joint utilization.*

## Conventional assessment

Conventional standardized clinical measures such as the Fugl-Meyer Motor Assessment [16] and Chedoke-McMaster Stroke Assessment [17] attempt to quantify movement impairments resultant from abnormal flexion synergy through observational analysis. Furthermore, observational analysis is then scored via nominal or ordinal scales. These evaluation tools have adequate psychometric properties, granted the clinician is properly trained in administration. However, they lack quantitative control and measurement with ratio-level data that arguably offers more meaningful and higher resolution information. Robotic methods of movement analysis can provide the much-need higher resolution measurements of the impact of loss of independent joint control on reaching performance [14] and hand function. Such methods offer both the researcher and clinician more powerful information for investigating and diagnosing movement problems, their underlying mechanisms, and response to intervention. The following section discusses a series of robotic methods for measuring the effect of loss of independent joint control on reaching and hand function.

## How do you measure/quantify the effect of loss of independent joint control on reaching and hand function?

The dynamic nature of the expression of flexion synergy and subsequent loss of independent joint control is best quantified using rehabilitation robotic devices. For example, reaching range of motion (work area) monotonically decreases as a function of increasing abduction load [15]. Conventional rehabilitation practice stands to benefit greatly from a quantitative evaluation of movement that directly measures the effects of loss of independent joint control in the context of movement [14]. While there are several electromyographic studies that have reported abnormal muscle synergies in the context of muscle activation patterns [18–20], we focus here on the application of robotics to quantifying the impact on reaching movement as this is the most relevant to enhancing movement problem diagnosis and development of targeted interventions in stroke rehabilitation. It should be noted that other quantitative robotic methods have been reported for quantifying the effects of abnormal muscle co-activations on reaching movements such as circle drawing [21] and outward reaching [22], however these methods are limited to measurement of reaching without the requirement of shoulder abduction torque generation. In other words, reaching movements occurred along a horizontal support surface where the participant was not required to lift the arm up off of the surface during task performance. While reaching along a horizontal support surface may still reflect the constraints of loss of independent joint control, they do not capture the dynamic/progressive expression of loss of independent joint control when the individual with stroke is required to actively elevate and maintain the arm above the support surface under increasing amounts of required shoulder abduction torque. Critical for clinical evaluation is that the dynamic expression of loss of independent joint control varies amongst individuals with stroke and appears to relate to the level of motor system insult severity [14].

### A system for measurement and overview of paradigms

A series of robotic metrics utilizing the ACT^3D^ are described illustrating the quantification of the effect of loss of independent joint control on reaching performance and hand function. Following the section below on “reaching work area,” suggestions are made for commercially available mechatronic devices that may be capable of administering these specific paradigms. But, for a recent exhaustive survey of existing mechatronic devices utilized in laboratories around the world, please see Maciejasz et al. [23].

The following methods decrease in their complexity offering appropriate solutions required across the translational spectrum of laboratory to clinical practice. Importantly, these methods are all capable of addressing the dynamic nature of loss of independent joint control in that its expression is increased as a function of proximal joint requirements (shoulder abduction). The method for measuring “*maximum shoulder abduction*” is described first as its magnitude is utilized in all subsequent robotic paradigms to standardize and normalize abduction loading. The proceeding sections discuss the series of robotic measures for quantifying the effect of loss of independent joint control on reaching and hand function. The first section begins with the most comprehensive and robust metric, *“reaching work area,”* which quantifies the total reaching workspace of the paretic arm at various abduction loads up to and beyond the weight of the limb [15]. This metric has been validated [14] and utilized as a clinical trial outcome measure [24, 25] demonstrating responsiveness to change. It has the capacity to capture range of motion deficits in all components of the horizontal workspace of the arm, and most importantly, at all functional abduction loading abilities of the individual. The second section introduces a reduced metric, *“reaching distance,”* that quantifies reaching distance at the same abduction loads. This metric reduces the data acquisition and implementation time by focusing on the region of workspace directly in front of the participant but still has the capacity to capture range of motion deficits at all functional abduction loading abilities of the individual. Due to the decreased movement trial time of this metric, in the laboratory setting, this protocol may be implemented in combination with acquisition of other data requiring large numbers of repetitions with little impact on the participant/patient. The third section introduces the most efficient and therefore clinically viable metric, *“maximum reaching abduction load (MRAL*_*near, far*_ *),”* that quantifies the abduction load at two standardized reaching distances (near and far). This metric boils the prior two methods down to representing the thresholds at which the loss of independent joint control impairment first emerges impacting full reaching range of motion (far target) followed by when it overtakes and eliminates volitional reaching ability (near target). It represents the most efficient quantitative metric of shoulder/elbow coordination and can be completed in ~15 min. Finally, the fourth section discusses *“maximum hand opening and closing”* at terminal reaching distance under various abduction loads. Hand function deteriorates as a function of increasing abduction loading [26]. This method accounts for the deterioration of hand function as a result of abduction loading as well as from the additive demands of reaching outward.

### Measuring maximum voluntary abduction torque

Obtaining the maximum voluntary joint torque for shoulder abduction is required when quantifying the effect of loss of independent joint control on reaching and hand function. This is critical so that changes in strength/weakness are taken into consideration as the effect of loss of independent joint control is evaluated. These two motor system impairments may follow independent recovery trajectories and therefore can confound the measurement if not accounted for. Additionally, maximum voluntary torque for shoulder abduction is measured in order to standardize the abduction loading values to a physiological magnitude for all of the ACT^3D^ protocols described. The evaluation of maximum voluntary torque can be quantified by any mechatronic device capable of maintaining the arm/hand static and accurately measuring isometric shoulder abduction joint torque.

#### Experimental setup

**Fig. 1:**
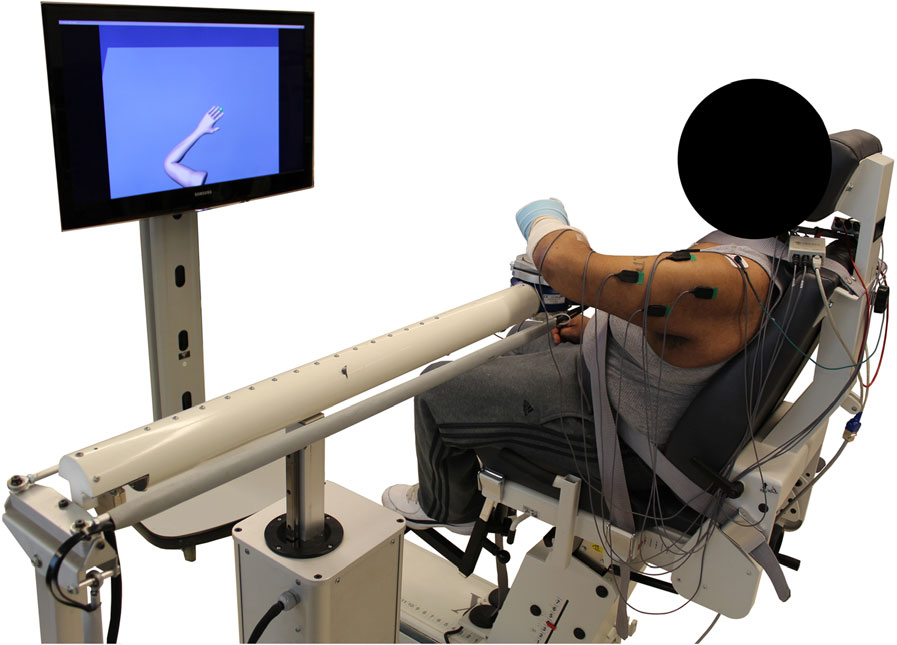
Participant setup in the ACT^3D^

Participants sit in a seating and positioning system (Biodex3 track and chair) with their arm resting in a forearm-hand orthosis attached to the ACT^3D^ (Fig. 1). The orthosis maintains the wrist and hand in a neutral position and the participant’s trunk is immobilized to prevent unwanted compensatory movements by a set of straps attached to the chair. The shoulder is positioned with the upper arm perpendicular or 90° to the line of gravity when the arm is resting on a haptically rendered horizontal table (virtual table maintained by the device and displayed in visual feedback). Additionally, the participant’s upper arm is positioned 40° anterior to the anatomical frontal plane (clinically known as “horizontal shoulder adduction”) and the elbow is placed in a 70° elbow angle. This position will be referred to as the “home position” in subsequent protocols. The standardized home position, in combination with measured limb segment lengths, is utilized by the ACT^3D^ software to calibrate a graphic representation of the arm and illustrate it on a computer screen in front of the participant.

#### Experimental protocol

Multiple repetitions of maximum shoulder abduction are completed until 3 values are obtained that are within 10 % of each other without the last repetition being the greatest [12]. During the measurement, joint torque is measured and displayed in real-time in the form of a rising bar graph while the participant attempts to maximize shoulder abduction torque. Following each repetition, the maximum value is marked and the participant is encouraged to exceed it on the next attempt. Additionally, the gain of the feedback display is reduced by 10 % without the participant’s knowledge in order to maximize repeated efforts. Data collection for each trial is 5 s and peak shoulder abduction torque is displayed to the experimenter following each trial.

### Reaching work area- *a comprehensive and validated robotic measure of reaching workspace as a function of increasing abduction loading*

Work area is a quantitative measurement of motor impairment (combined shoulder-elbow active range of motion) performed in a functional context (multiple abduction loads). It is administrated in a standardized fashion and utilizes 3D kinematic and kinetic analyses as opposed to subjective interpretations of movement and therefore, leaves little room for experimenter or clinician bias [15]. The quantitative measurement has been cross-validated with qualitative clinical assessments of impairment, activity and participation limitation and has been shown to augment conventional clinical evaluation of upper extremity function by specifically identifying the impact of loss of independent joint control on functional reaching [14]. Work area has been successfully implemented as a primary outcome measure in previous work attempting to demonstrate the amelioration of reaching function through the reduction of loss of independent joint control impairment [24, 25].

#### Experimental setup

See the experimental setup paragraph of the maximum abduction torque method above.

#### Experimental protocol

Following setup of the participant in the device, the optimal chair-robot orientation is determined such that the maximum available workspace can be evaluated. The position is determined by rotating the participant’s chair in relation to the ACT^3D^ and passively moving the participant’s arm throughout the workspace in order to identify the optimal chair-robot orientation.

**Fig. 2:**
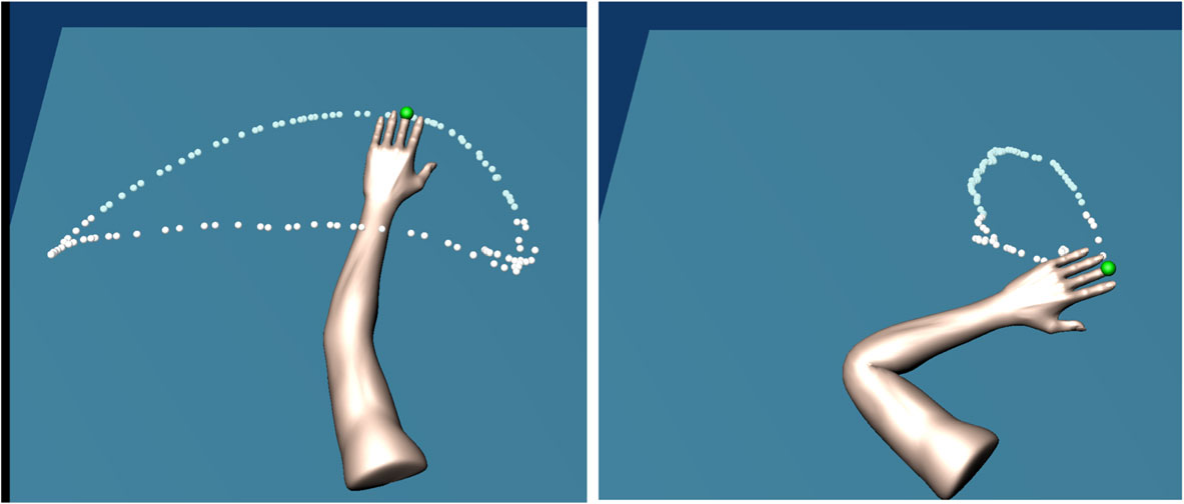
Real-time graphical display of work area trajectory during a trial on the haptic horizontal table (left) and at 50 % abduction loading (right)

During work area measurement, participants are asked to move the tip of their hand in a circular motion producing the largest envelope possible with their paretic arm while it is fully supported by and gliding on the horizontal haptic table. The task begins by the participant acquiring the home position while supported by the haptic table. Once the home position is acquired, data collection is initiated sampling at 60 Hz and is indicated to the participant by a visual signal of the home target disappearing. The task is performed slowly to minimize the effects of hyperactive stretch reflexes or spasticity at the elbow and shoulder joints. Participants perform the task in both the clockwise and counterclockwise directions in order to acquire the full range of motion. While pilot data suggests movement in the clockwise direction only is sufficient to capture the complete range of motion for right-affected individuals and counterclockwise for left-affected individuals, it has not been fully validated [27]. Real-time performance feedback is provided in the graphical display by dropping white dots along the endpoint trajectory (Fig. 2).

**Fig. 3:**
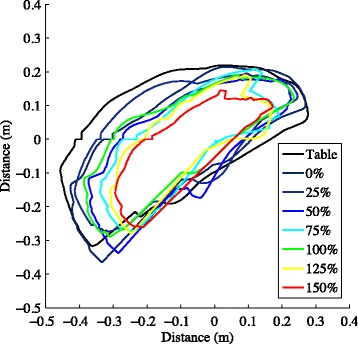
Example work areas (right-affected)

Since the work area measurement attempts to capture the total available reaching range of motion, envelopes generated from a minimum of 3 trials in each direction are superimposed and the area of the combined envelope is calculated [15]. One minute of rest is given between each 15-s trial to eliminate fatigue, and verbal feedback is given in every attempt to encourage the participant to achieve the maximum movement excursion while moving slowly. Following completion of the work area performed while supported by the haptic table, the haptic table is lowered using controls on a MATLAB graphic user interface by approximately 10 cm. In subsequent trials participants are required to actively support their arm just above the horizontal haptic table resulting in 90° of shoulder abduction/elevation as it was when supported by the original haptic table. Participants are then instructed to maintain the hand close to the center of their body prior to lifting the arm. This is a critical aspect of the measure as work area declines toward zero (close by the center of the body) at the heaviest abduction loads. Once the arm is lifted from the haptic table, data collection begins and a deterrent change in background color occurs any time the participant’s arm inadvertently deflects off or intentionally rests upon the haptic surface. The participant is given regular and immediate verbal feedback and encouragement to maximize reaching work area without dropping the arm down onto the haptic surface. The protocol is repeated while the ACT^3D^ provides forces along its vertical axis to alter the amount of abduction loading that the participant is required to support. A total of 4 to 9 abduction loading levels are utilized, including on the haptic table, and are randomized for testing (see Fig. 3 for an example of work area used with permission [24]). Abduction loading levels represent percentages of the participant’s maximum isometric shoulder abduction strength including but not limited to 0, 12.5, 25, 37.5, 50, 62.5, 75, 87.5, and 100 % of maximum voluntary torque for isometric shoulder abduction. This allows for a standardized protocol grounded to the maximum physiological motor output and presumably the maximum expression of the motor impairment. Prior work has also provided loading as a percentage of limb weight to prioritize a functional standardization over a physiological standardization.

#### Other devices

There are a few commercially available devices capable of measuring work area but would require a modification or the concurrent use of supportive equipment to be effective. The most promising device for quantifying work area is the ArmeoPower (Hocoma AG, Switzerland). The device allows for dynamic reaching movements and is used to assist reaching movements in predefined trajectories. Restricting the ArmeoPower to horizontal plane motion at shoulder height, freeing outward motion, and emulating abduction loading may allow the device to systematically quantify work area under progressive abduction loads. A second commercially available device is the SaeboMAS (Saebo Inc., Charlotte, NC). The SaeboMas provides analogue unweighting of the arm. It could be utilized to quantify work area however it can not provide additional abduction loading (or weighting) like the ACT^3D^ limiting its ability to measure reaching performance under functionally relevant abduction loads. Additionally, the SaeboMAS is not instrumented so it would need to be used concurrently with a 3D motion analysis system in order to measure movement kinematics. A final device that is instrumented and capable of quantifying work area is the InMotionARM Interactive Therapy System (Bionik, Watertown, MA). This device restricts movement to the horizontal plane however does not allow dynamic motion in abduction like the ACT^3D^. Therefore, while the system can quantify reaching work area while supported on a horizontal surface, it is unable to quantify the detrimental effects of abduction loading on reaching performance. This device could possibly still be utilized through a modification that controlled/measured isometric abduction forces during the reaching effort. Each of these three commercially available devices can be used to quantify work area but only in part. In fact, all of the protocols described in subsequent sections could be carried out in part by ArmeoPower, SaeboMas, and InMotionARM but only with modifications. Therefore, they will not be reiterated in subsequent sections. Due to the difficulty of such modifications, perhaps the most effective approach would be to bring a device like the ACT^3D^ to market in order to best quantify the effect of abduction loading on the loss of independent joint control outside of the laboratory environment.

### Reaching distance- *a reduced method measuring outward reaching distance as a function of increasing abduction loading*

Reaching distance reduces the measurement of total reaching workspace of the arm down to a metric of endpoint reaching trajectory distance to an outward target. This metric is based on original dynamic reaching investigations of supported and unsupported reaching movements that demonstrated a reduction in outward reaching distance when reaching against gravity compared to while sliding along a frictionless table [7]. It has since been extended to include the standardized abduction loads described in the work area paradigm and used as the primary outcome for investigations of progressive abduction loading therapy [28]. Analysis of the minimal detectable change score for reaching distance has been presented in abstract form [29].

The reaching target is standardized to a shoulder and elbow joint configuration such that the participant is reaching nearly to their end range of motion directly in front of the shoulder. The measurement of reaching distance from the home position to the furthest point toward the reaching target captures the maximum combined shoulder and elbow joint excursion in the most functionally relevant direction. This reduced method for quantifying loss of independent joint control affords a unique benefit to scientific investigations. For example, investigations that require a movement task that can be repeated many if not hundreds of times are appropriate for the metric of reaching distance as opposed to reaching work area since it can be administered in much less time and therefore for a high number of repetitions. Overall, the metric represents an efficient and functionally relevant kinematic and kinetic evaluation of the effect of loss of independent joint control on reaching performance.

#### Experimental setup

See the experimental setup paragraph of the maximum abduction torque method above.

#### Experimental protocol

**Fig. 4:**
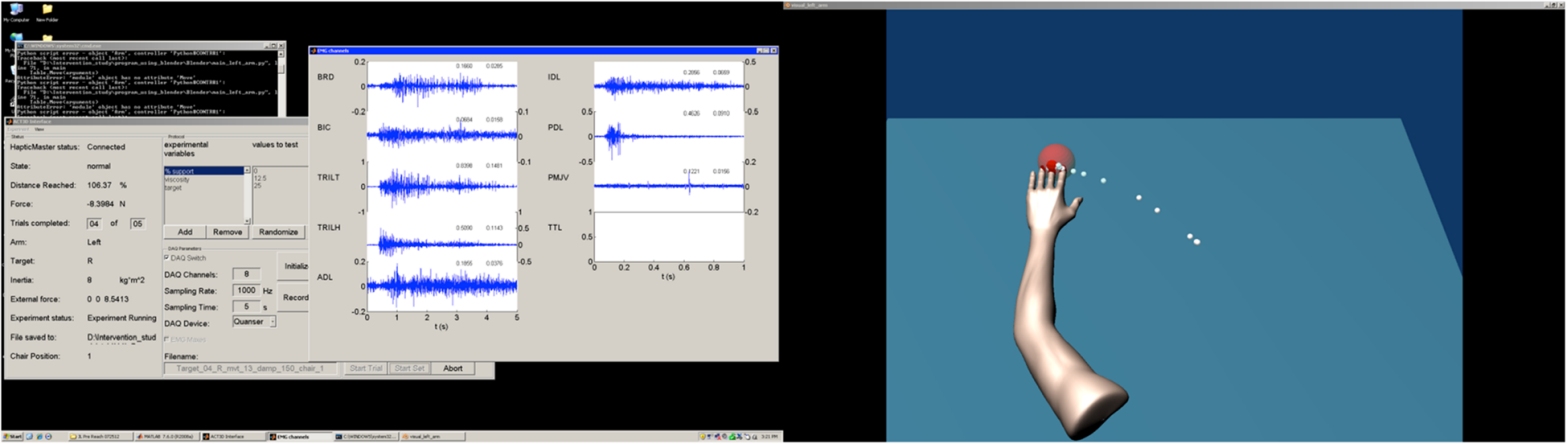
Real-time graphical display of one reaching distance trial (right) paired with EMG acquisition (left)

**Fig. 5:**
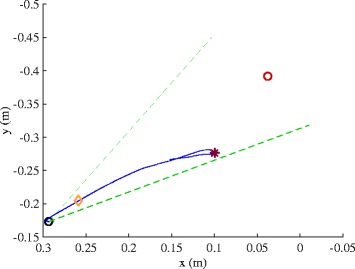
Example of a reaching distance trial at 50 % of maximum voluntary torque for shoulder abduction. The participant’s maximum reaching distance noted by the red asterisk is short of the reaching target indicating the impact of loss of independent joint control on reaching range of motion

Once positioned and supported by the haptic table, participants are asked to view the feedback monitor and slide their hand along the table acquiring the home position. After the endpoint of the hand acquires the home position, data collection begins by the ACT^3D^. One second after data collection is initiated, a second circle representing the movement target appears on the screen as a cue for the participant to begin the movement (red sphere in Fig. 4). The movement target is located requiring an additional 90° of elbow extension and 40° of shoulder flexion from the home position to acquire. This location lies approximately in front of the participant’s shoulder with the arm nearly fully extended (10° short of full elbow extension). Participants are instructed to move as far and as fast as possible toward the target and then maintain the final position until the target disappears (end of data collection). To avoid anticipation, it is stressed in the instructions that the participant does not react to the appearance of the target but instead begin the movement at his/her discretion within a time window of 2 s. Rapid (ballistic) movements are strongly encouraged through verbal cuing of the experimenter prior to and during every repetition. Feedback on performance is also given intermittently to maximize performance and expedite the protocol. Realistic visual feedback of movement performance is also provide by the avatar of the participant’s arm (Fig. 4) that emulates movement in real-time. Additionally, during and slightly following the completion of each target reach, the hand path is displayed to the participant. The length of data collection is 5 s per trial. Five consecutive repetitions are conventionally performed with the goal of identifying the maximum reaching distance (Fig. 5).

Participants repeat the reaching movements for standardized levels of shoulder abduction loading based on the needs of the investigation or clinical evaluation. During abduction loading trials, participants are required to lift the arm off of the haptic table prior to acquiring the home position. A ballistic reach is made to the same outward target but is performed while the arm is maintained elevated above the haptic table. Conventionally, one set of 5 repetitions is performed for each abduction loading condition and one set while supported on a horizontal haptic surface (always performed first). The abduction loading conditions include but are not limited to 0, 12.5, 25, 37.5, 50, 62.5, 75, 87.5, and 100 % of maximum voluntary torque for isometric shoulder abduction. In order to prevent fatigue a 15 s rest is required between repetitions and a 3-min rest is required between each set.

### Maximum reaching abduction load (MRAL_near, far_)- *an efficient and clinically relevant metric*

During the process of recovery from stroke, the expression of loss of independent joint control varies in its onset and progression for each patient. Furthermore, in acute stroke and clinical practice in general, there is a substantial time constraint obviating lengthy evaluations. Therefore, a clinically viable metric must be expeditious and not suffer floor and ceiling effects for a stroke population varying in severity. To address these limitations, we have recently developed the MRAL_near, far_ that identifies two distinct thresholds in a time-efficient and therefore clinically-viable fashion. The method is exceptionally fast since it employs a binary decision tree algorithm to optimally determine the threshold abduction loads. While formal validation has not yet been completed, preliminary analysis of its validity has been completed and presented in abstract form [30]. The thresholds identified by the metric are as follows; first, the threshold at which loss of independent joint control overtakes and eliminates reaching function (MRAL_near_), and second, the threshold at which it just begins to impact reaching function (MRAL_far_). Identification of both thresholds eliminates the limitation of floor/ceiling effects. For example, severe expression of loss of independent joint control would suffer a floor effect of the MRAL_far_ threshold in that the abduction loading level would approach 0 %, therefore the MRAL_near_ threshold would best capture the detrimental impact on reaching function in the more severely affected patient. In the opposite case of very mild expression of loss of independent joint control, there would be a ceiling effect in the MRAL_near_ threshold in that the abduction loading level would approach 100 %, therefore the MRAL_far_ threshold would best capture the impact on reaching function still existent but only at more demanding efforts in the mildly affected patient.

#### Experimental setup

See the experimental setup paragraph of the maximum abduction torque method above.

#### Experimental protocol

Once positioned and supported by the haptic table, participants are asked to view the feedback monitor and slide their hand along the table acquiring the target to familiarize themselves with the avatar feedback (Figs. 2 and 4). The haptic table is then lowered 10 cm and a load of 25 % of maximum voluntary torque for isometric shoulder abduction is rendered by the device. The participant is instructed to begin with the arm close by the center of the body or just behind the home position and then attempt to lift the arm up and reach toward the target. Because the home position is so close to the center of the body, the distance of this reach is considered negligible and therefore represents a reaching distance of “zero” and is defined as the “near target.” If the home target is acquired, it disappears and the trial is a success. A set of 10 repetitions is completed for learning purposes at the 25 % level and can be adjusted by the clinician but should represent an “easy” effort.

Next, a binary decision tree algorithm is used to identify the maximum reaching load, or the highest load at which the participant can successfully reach the target. The algorithm begins by starting at a 50 % load. The next load is increased or decreased by 50 % of the remaining load range depending on the success or failure of the condition respectively. For each condition, the participant is given up to 3 attempts to be successful with a mandatory 1-min rest following a failed attempt. If the condition is successful the next [larger] load is attempted following a mandatory 1-min rest. In contrast, if the condition is failed, the next [smaller] load will be attempted following a longer 3-min rest. In summary, using the binary decision tree algorithm, the maximum reaching load can be determined very rapidly in 5 steps achieving a measurement resolution to the nearest 3.125 % of maximum abduction strength. Therefore the MRAL_near_ described here represents the threshold at which the loss of independent joint control overtakes and eliminates reaching function.

This procedure is then followed for the “far target” representing full reaching range of motion. The only difference in procedure is that the target that the participant is required to reach for is 10° short of full elbow extension and 70° of shoulder flexion (from the coronal plane). The MRAL_far_ therefore represents the threshold at which loss of independent joint control begins to impact reaching function.

### Maximum hand opening (pentagon area) and closing (grip force)- *a comprehensive kinematic and kinetic measure of hand function during abduction loading and reaching*

It is well known that hand function is greatly impaired following stroke. Clinical assessments that evaluate hand function often involve reaching and manipulation of objects such as in the action research arm test [31]. In addition to not being kinematicaly quantitative, a primary limitation of these types of assessments is that they involve reaching against only one abduction load (gravity) and therefore don’t address the progressive impact of loss of independent joint control on hand function. For example, abnormal flexion of the hand increases as a function of increasing abduction loads [26]. Furthermore, volitional extension of the thumb and fingers is reduced as a function of increasing abduction loads during reaching [32]. Here we present a kinematic/kinetic measurement of maximum hand opening (pentagon area) and grip force as a function of abduction loading to provide a quantitative measurement of the impact of loss of independent joint control on hand function. Formal validation of this method is not yet published but still offered as a logical extension of techniques for quantifying hand function during reaching under progressive abduction loads.

#### Experimental setup

**Fig. 6:**
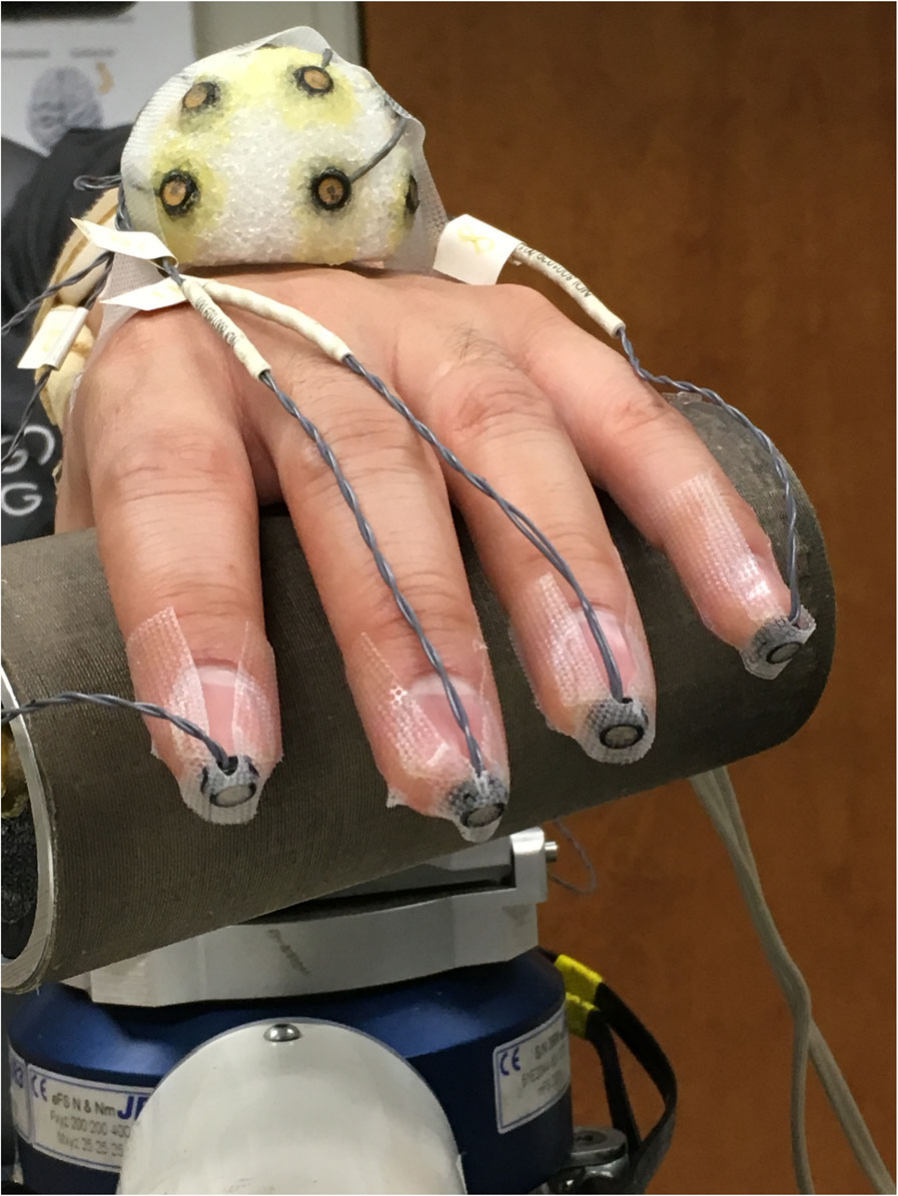
Hand mounted to the cylindrical force mat with motion analysis markers in place. Forearm is securely mounted to the ACT^3D^ for the manipulation of abduction loading during reaching movement

The participant is setup in the ACT^3D^ as described in the experimental setup paragraph of the maximum abduction torque method above with the addition of a custom cylindrical force sensor and 3D motion analysis markers for measuring hand opening and closing respectively (Fig. 6). The hand mount and forearm orthosis are rigidly attached to the ACT^3D^. The pressure sensor mat (Pressure Profile System Inc., Los Angeles, CA, USA) allows real-time pressure measurement under the digits during hand grasping. Infrared light-emitting diode markers allow kinematic data to be collected using two Optotrak camera systems (Optotrak 3020 and Certus, Northern Digital Inc., Waterloo, ON, Canada) from all digits during hand opening. Alternatively, assessment of hand kinematics may also be measured using inertial and magnetic sensors [33].

#### Experimental protocol

Once positioned and supported by the haptic table, participants are asked to view the feedback monitor and slide their hand along the table acquiring the home position to familiarize them with the avatar feedback. Once oriented, the participant is asked to reach outward as far as they can to a standardized distant target (same distant target as described in the reaching distance and MRAL_far_). Once the participant reaches to their maximum ability they are asked to either lift the arm off of the haptic surface or retain it on the surface while maintaining their reaching position for 2 s. Next, they are instructed to either maximally open or close the hand without disengaging the reaching task and maintain the effort for at least 3 s. Combinations of rest vs. lift of the arm and open vs. close of the hand are randomized with each performed for 3–5 repetitions. During the arm lifting conditions, abduction loading is administered at 25 and 50 % of maximum voluntary abduction torque.

**Fig. 7:**
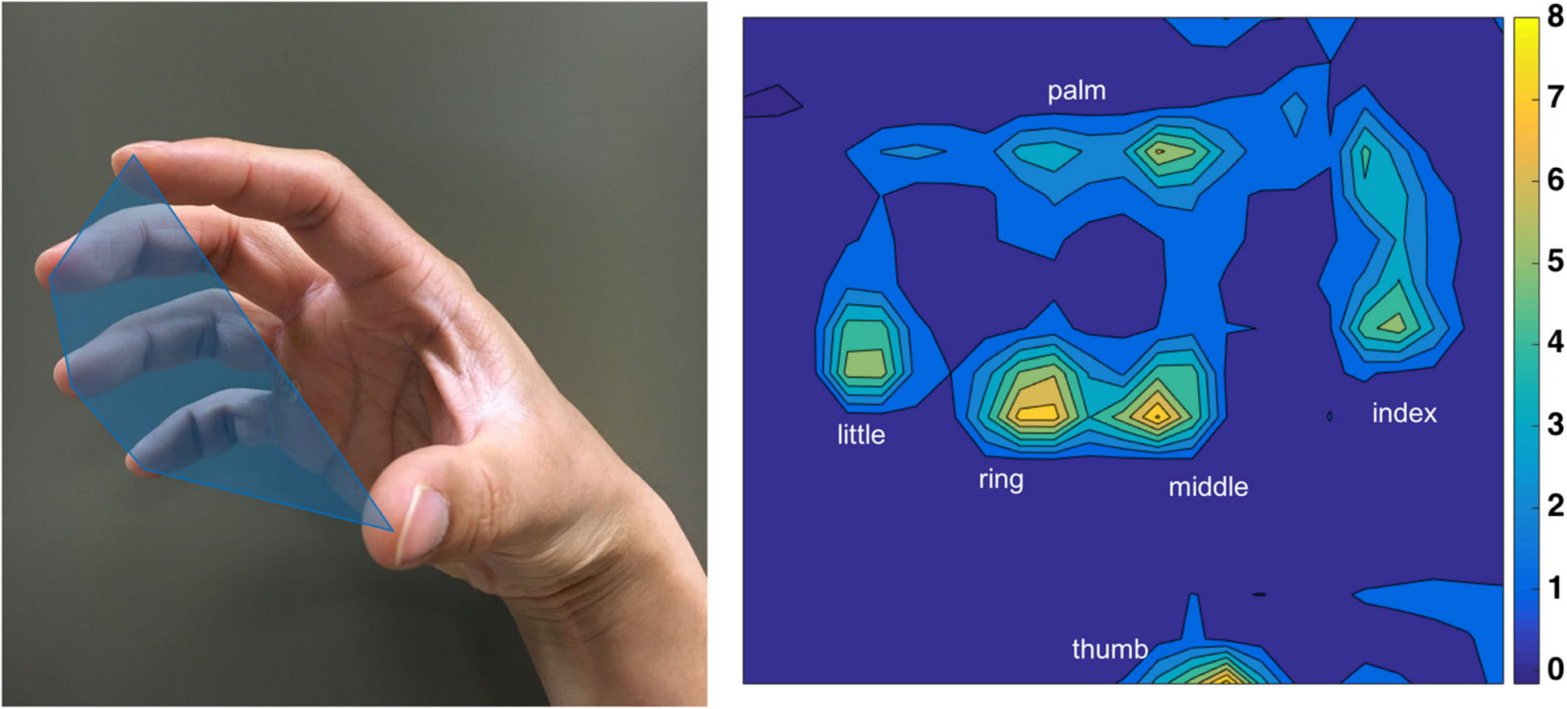
Hand pentagon area is calculated by measuring the area obtained by connecting the tips of all digits from kinematic data (left). Grasping force heat map obtained from the custom force mat (right)

Maximum hand opening is measured by calculating the hand pentagon area obtained from the motion analysis markers (Fig. 7). The hand pentagon area is normalized to the pentagon area of the non-paretic hand in each participant with the hand flat on a table. Grasping force is measured first at the end of the reach (labeled as synergy-induced grasping force) and during the volitional attempt to maximally grasp while maintaining the reach (labeled as total grasping force). Total grasping force is calculated as the sum of forces generated by the digits averaged over the 3 s grasping effort (Fig. 7).

## Recommendations for measurement of the loss of independent joint control

Both the scientist and the clinician desire an accurate quantitative evaluation of the loss of independent joint control. Choosing the appropriate measure for quantifying the effect of loss of independent joint control on reaching and hand function is dependent upon pragmatic constraints. For example, in the clinical environment, scheduling and the patient’s medical state/tolerance both limit the time that may be spent in evaluation. The expedited methods of MRAL_near/far_ or maximum hand opening will be the measurement of choice for proximal shoulder/elbow or hand loss of independent joint control respectively for the clinical environment. In contrast, in the research laboratory a more detailed investigation of loss of independent joint control may be required. In that case, recruitment would need to select for participants that can tolerate a longer evaluation. With a remaining need for experimental efficiency, the measure of reaching distance at several abduction loads may be ideal. Overall, the most critical requirement of all of the paradigms is that reaching movement and hand function must be measured under controlled abduction loading conditions. Measurement under abduction loading conditions allows for the direct quantification of loss of independent joint control and defines with great resolution how the motor system impairment impacts natural arm and hand function. Furthermore, the inclusion of abduction loads that go beyond limb weight will reflect the full functional impact of loading that occurs in real-world arm use such as when transporting an object [14].

## Conclusions

Clinicians and scientists will greatly benefit from employing paradigms described in this review of robotic methods for quantifying the loss of independent joint control. In the clinic, the high-resolution measurements will afford the ability to better target the impairment and track recovery [24, 25, 34]. In the laboratory, high resolution measurements will facilitate the elucidation of underlying neural mechanisms of the loss of independent joint control [15] and subsequently catalyze the development of novel impairment-based therapies designed to directly target this cardinal motor impairment of stroke [28].

## Abbreviations

ACT^3D^: Arm coordination training three dimensional robotic device
MRAL_near/far_: Maximum reaching abduction load (near & far targets)
